# A rare case of invasive sinonasal carcinosarcoma

**DOI:** 10.1016/j.ijscr.2020.04.072

**Published:** 2020-05-07

**Authors:** Eduardo Luis de Souza Cruz, Antonia Taiane Lopes de Moraes, Francisco de Souza Neves Filho, José Thiers Carneiro Junior, Bruno Thiago Cruz e Silva, Victor Angelo Martins Montalli, Sérgio de Melo Alves Júnior, João de Jesus Viana Pinheiro

**Affiliations:** a School of Dentistry, Federal University of Pará (UFPA), Belém, PA, Brazil; b Department of Oral and Maxillofacial Surgery at Ophir Loyola Hospital, Belém, PA, Brazil; c Department of Oral Pathology, São Leopoldo Mandic, Institute and Research Center, Campinas, São Paulo, Brazil

**Keywords:** Carcinosarcoma, Nasal cavity, Prognosis, Aggression, Pathology, Neoplasms, Case report

## Abstract

**Introduction:**

Carcinosarcomas are rare malignant neoplasms formed by embryonic tissues (ectoderm and mesoderm) and present remarkably aggressive character with unfavourable prognosis. These lesions are rarely diagnosed in the sinonasal cavity and only a few cases are reported in the literature. This manuscript aimed to report a rare case of a large and aggressive sinonasal carcinosarcoma that involved the facial middle third and to discuss the proposed treatment.

**Presentation of case:**

A 54-year-old female patient sought treatment for a large swelling on the left side of the face. A CT-scan revealed an expansive hypodense image in the facial middle third. An incisional biopsy was performed under local anaesthesia and the material was sent for histopathological analysis.

**Discussion:**

Due to the extremely aggressive character of carcinosarcomas, the optimal management of carcinosarcomas remains uncertain and a challenge for clinicians. In this case report, the prognosis of such a large tumour became unfavourable since the patient sought late for treatment. Therefore, suspicion and early detection are crucial for improving the prognosis and quality of life of the patients with such neoplasia.

**Conclusion:**

Besides being rare and extremely aggressive, sinonasal carcinosarcomas present worse patient survival when compared to other carcinosarcomas in the head and neck region. Thus, this report may contribute to a better understanding of this tumour behaviour.

## Highlights

• This report presents a rare case of injury from two embryonic leaflets, located in the sinonasal cavity.
• Clinical, radiographic, histopathological and immunohistochemical panel data were used to obtain the diagnosis.
• The treatment modality employed was the left maxillectomy through Weber-Ferguson subpalpebral surgical access.
• After surgery, the patient presented remission of some signs and symptoms and was referred to the oncology department.

## 1 Introduction

Carcinosarcomas are extremely malignant neoplasms composed of an epithelial component represented by focal squamous cell and a mesenchymal component that corresponds to sarcomatoid stroma [[1], [2], [3], [4], [5]]. These remarkably aggressive lesions are usually diagnosed in males and females between the 6th and 7th decade of life and present a fast and symptomatic progression with unfavourable prognosis [[6], [7], [8]].

Carcinosarcomas may arise from any squamous epithelium of the body, such as salivary glands, respiratory tract, upper aerodigestive tract or even female reproductive organs; however, its occurrence in the sinonasal region is extremely rare and only a few cases have been reported [[1], [2], [3],^8^]. This manuscript aimed to report, following SCARE criteria [9], a rare and invasive case of a large carcinosarcoma that involved the facial middle third.

## 2 Case report

A 54-year-old brown-skinned female presented to the Bucco-maxillofacial Surgery and Traumatology Department with a large swelling on the left side of the face. The patient reported progressive symptoms over the last 3 years such as dystopia, dysphagia, trismus, respiratory obstruction and dysphonia.

**Fig. 1:**
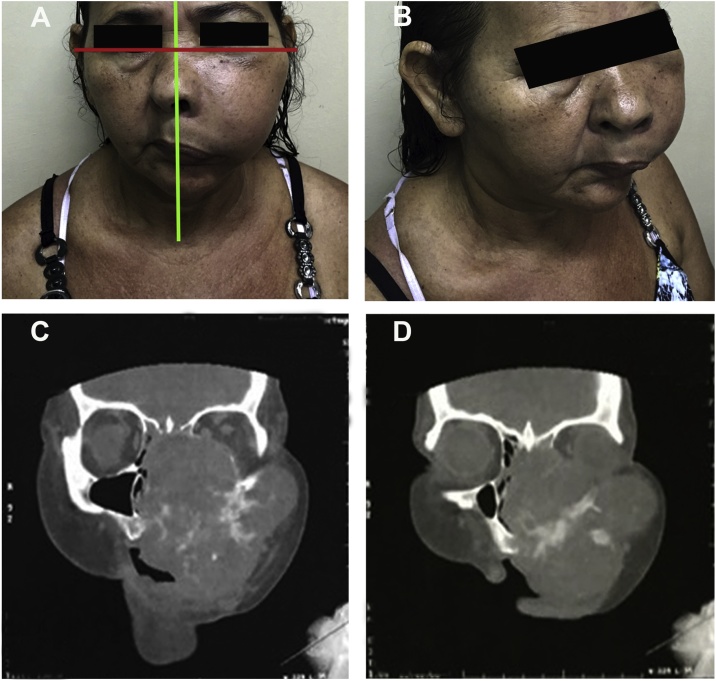
Clinical extraoral photograph at the beginning of treatment and Pre-treatment multislice computed tomography. In 1A is observed facial asymmetry. In 1B is observed in the maxilla region between the zygomatic arch and the left labial commissure the extent of the tumor that promotes dystopia and nasolabial fold deletion with right nose displacement. In 1C and 1D is observed a large expansive hypodense image involving the middle third of the face.

The extraoral clinical examination of the maxilla revealed a hardened and symptomatic tumour located between the zygomatic arch and the labial commissure, dystopia and depletion of the nasolabial fold with the displacement of the right nostril (Fig. 1A and B), while no palpable lymph nodes were observed. The patient denied smoking, alcoholism, previous radiotherapy and family history of concomitant diseases. The intraoral examination showed an increased volume in the left posterior palate with hardened consistency, normal colouration, absence of ulceration, erasure of the vestibular sulcus and sensitivity to touch.

A multislice CT-scan revealed an expansive hypodense image in the facial middle third that involved hard palate, left zygoma, ethmoid and sphenoid, invasion and destruction of the nasal cavity, total obstruction of the nasopharynx and partial destruction of the orbit. The lesion was poorly circumscribed, which is suggestive of a malignant neoplasm with extensive bone destruction and involvement of structures near the base of the skull such as the ethmoid and sphenoid bones (Fig. 1C and D).

**Fig. 2:**
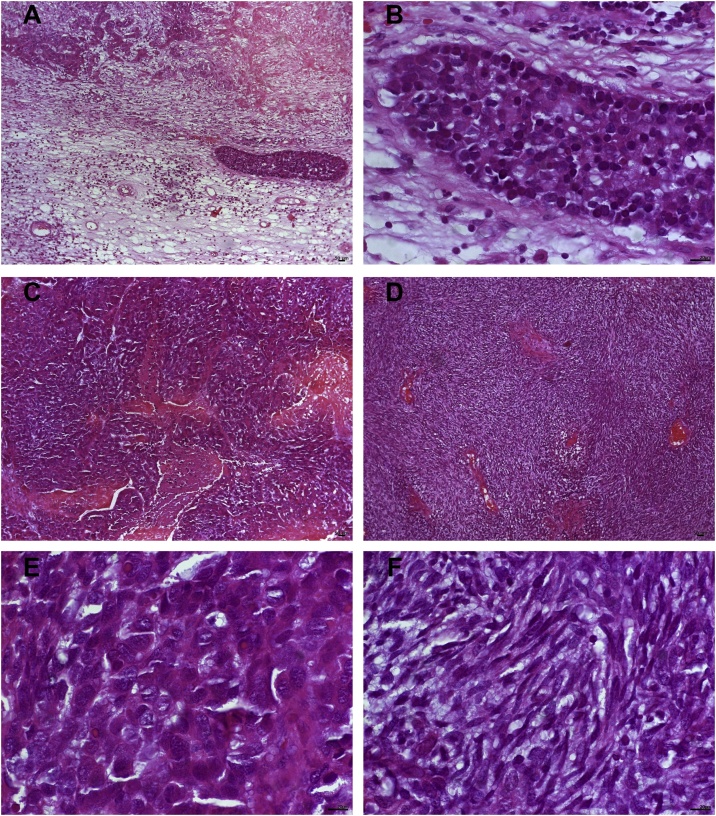
A–F. Histopathology features of the sinonasal carcinosarcoma in the incisional biopsy specimen showing proliferation of cuboid cells with nuclear and cytoplasmic pleomorphism. It was also possible to observe the appearance of the tumour from the respiratory epithelial tissue (stained with HE). Scale bars: A, 200 μm and B, 20 μm.

An incisional biopsy was performed under local anaesthesia. The anatomopathological analysis revealed fragments of neoplastic tissue, a proliferation of cuboid cells with nuclear and cytoplasmic pleomorphism, atypical mitoses and block, island and lobe arrangements. The tumour was also observed in the respiratory epithelial tissue with the presence of keratin beads, in situ focal carcinoma, necrotic areas and scarce stroma, bleeding and myiasis (Fig. 2).

**Fig. 3:**
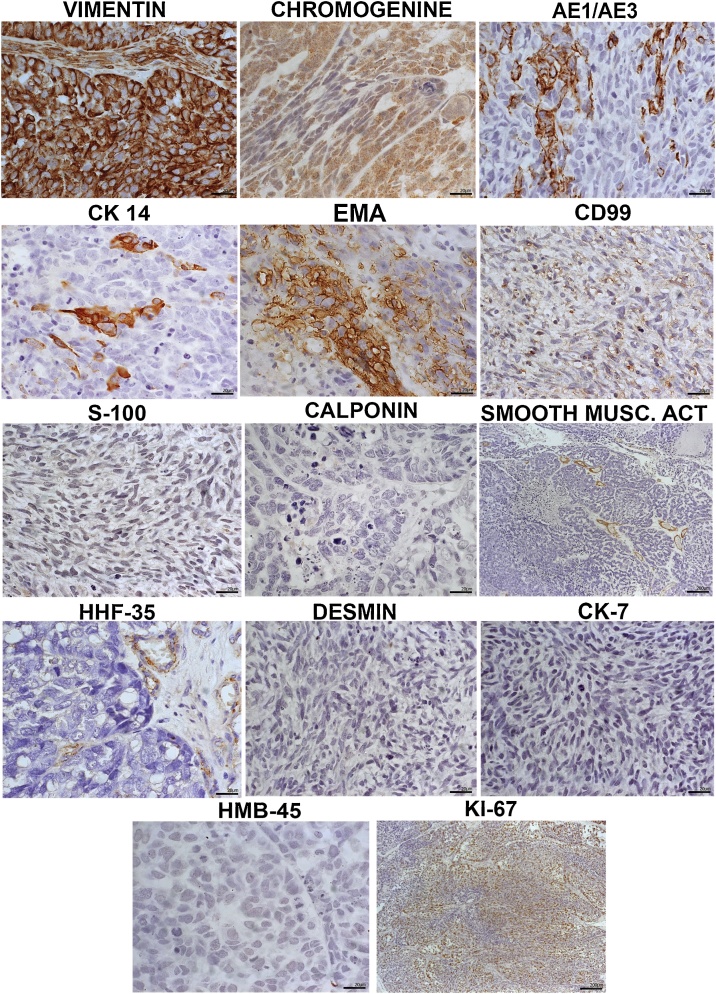
Immunohistochemical picture. Revealing diffuse positivity for vimentin and chromogranine. There was focal marking of AE1/AE3, cytokeratin 14, EMA and CD99. The neoplastic cells were negative for S-100, calponin, smooth muscle actin, HHF-35, desmin, cytokeratin 7 and HMB-45. About 80% of neoplastic cells were positive for Ki-67.

Moreover, the immunohistochemistry technique was used for diagnostic accuracy and revealed diffuse expression of vimentin and chromogranin, and focal marking of AE1/AE3, cytokeratin 14, EMA, and CD99. The neoplastic cells were negative for S-100, calponin, smooth muscle actin, HHF-35, desmin, cytokeratin 7 and HMB-45; while almost 80% were positive for Ki-67 (Fig. 3). All clinical, radiographic and histological features were suggestive of sinonasal carcinosarcoma.

**Fig. 4:**
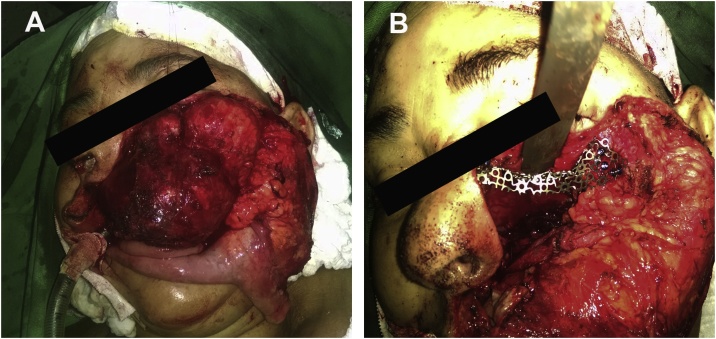
Surgical procedure. 5A, left maxillectomy. 5B, reconstruction of the orbit floor in titanium mesh.

The patient was submitted to partial maxillectomy with subpalpebral surgical access (Weber-Ferguson approach), albeit parts of the lesion were not removed due to large posterior extension. After the removal of a 11 × 6 × 4 cm single piece and curettage of the cavity, the orbital floor was reconstructed with titanium mesh (Fig. 4).

**Fig. 5:**
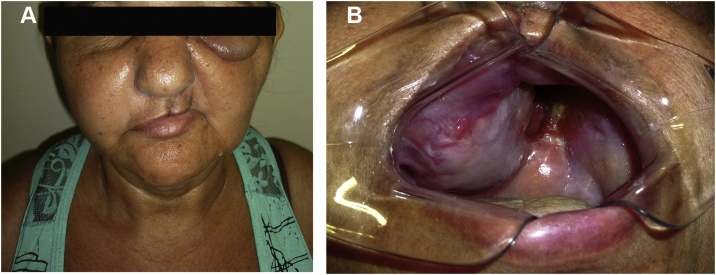
Control 30 days after the surgical procedure. 5A, Facial view. 5B, intra-oral view.

After 30 days surgical recovery, the patient presented remission of diplopia due to dystopia correction, the reestablishment of normal nasal breathing, no episodes of epistaxis, reduction of dysphagia, persistent dysphonia due to extensive loss of the hard palate and presence of a buccal fistula as a result of the partial maxillectomy (Fig. 5).

At the Oncology Department, the patient was submitted to 30 sessions of radiotherapy treatment (3.700MBq/100mCl) and followed intravenous chemotherapy with cisplatin at 8-h intervals during 3 months. Although clinical follow-ups were performed to control the extremely aggressive malignancy, the patient unfortunately died in 2019.

## 3 Discussion

A case of carcinosarcoma was diagnosed in a 54-year-old female after approximately 3 years of evolution. It was observed a large swelling in the face, extensive bone destruction near the base of the skull. The diagnosis was determined with the aid of the School of Dentistry of the Federal University of Par. The treatment protocol was a combination of surgery, radiotherapy and chemotherapy. Carcinosarcoma may arise from any squamous epithelium of the body, such as the respiratory tract, upper aerodigestive tract and genitals. In the head and neck area, this tumour most frequently occurs in salivary glands, larynx and pharynx [1,10,11]. As reported in this case, a highly invasive and destructive lesion only diagnosed at an advanced stage has unfavourable prognosis. Thus, the importance of early diagnosis remains crucial [12], since the sinonasal tumour is associated with severe clinical impairment [[13], [14], [15], [16]]. Notably, the symptoms of pain in the left maxillary sinus reported by the patient resembled chronic sinusitis. However, the patient was referred to the specialised service only after a gradual increase in facial pain and swelling, nasal obstruction and episodes of epistaxis over time.

The evolutionary stage of the lesion is extremely important, since early-diagnosed cases may result in a more favourable prognosis and lower mortality rates, while late-diagnosed cases are associated with more complex treatments and dismal prognosis. Thus, particularly for tumorus with great proliferative and invasive magnitudes, the initial clinical stage directly influences the treatment type. The patient here reported sought treatment after 3 years, which represents a significant period that resulted in a large lesion with the invasion of important anatomical sites and an unfavourable prognosis. It must be emphasised the importance of early detection of lesions that present a more aggressive behaviour such as sinonasal carcinosarcoma.

An epidemiological study conducted between 1973 and 2010 reported 135 cases of carcinosarcomas: 15 in sinonasal cavities, 34 in salivary glands and 81 in other regions of head and neck [6,7]. Among 15 cases reported in the literature that involved sinonasal cavities, five lesions were found in the maxillary sinus, one in the ethmoid sinus, one in the frontal sinus and one in the sphenoid sinus [4,1,8,10,13]. The case reported in this manuscript is of greatest relevance since this tumour is rarely found in the sinonasal cavity near to the base of the skull.

Although no specific symptoms are associated with sinonasal carcinosarcomas [2], all cases in the literature reported epistaxis and nasal obstruction [1,2,17], while an increase in volume is also a common sign [13,18]. Other symptoms such as hearing impairment and dysphagia may occur due to tumoural invasion of adjacent structures such as ear and pharynx, respectively [2,14,11,19]. Treatment of tumours with infiltrative and aggressive behaviour remains a great challenge since sinonasal regions are difficult to access. Concerning the case here reported, the neoplasia caused diplopia due to the eyeball displacement, dysphagia due to invasion of the nasopharyngeal space and trismus due to involvement of the masseter muscle. Considering the large size of the lesion and the uncertain pathological diagnosis, the patient was submitted to the surgical treatment so-called partial maxillectomy and subsequently followed by chemotherapy with cisplatin and radiotherapy, albeit a consensus on the treatment protocol is still needed.

Both treatment and prognosis of sinonasal carcinosarcomas are still not well established in the literature due to its rarity, tumourigenesis and uncertain clinical behaviour. Regarding histogenesis, carcinosarcomas present tumoural heterogeneity resulted from the presence of epithelial and mesenchymal components that constitute this mixed malignancy [1,2,4,20]. These lesions are histologically classified as part of a spectrum of sarcomatoid carcinomas. They consist of foci of overt carcinoma mixed with areas of divergent differentiation into mesenchymal tissues [12].

The pathogenesis of carcinosarcoma might be understood through studies on molecular genetics and immunohistochemical data of the disease stages. It is hypothesised that both monoclonal inactivation of the mechanism of tumour suppression on chromosome 17 and the block of p53 wild-type allele are the causes of this neoplasm. Ki-67 labelling is a common marker of the neoplasm stages, in addition to representing degrees of prognosis [21].

The reported immunohistochemical analysis revealed a diffuse expression of vimentin and chromogranin, which may be related to the degree of differentiation of epithelial and mesenchymal components. AE1/AE3 characterises the lesion as a carcinoma, excludes the possibility of a third component of neuroendocrine origin and, together with cytokeratin 14, EMA and CD99, makes it possible to identify the epithelial portion of the specimen and its degree of differentiation. The absence of S-100 and HMB-45 labelling precludes the diagnostic of melanoma, while the negativity for calponin, smooth muscle actin, HHF-35 and desmin demonstrates no other components of muscular origin. Cytokeratin 7 may be absent in some tumours such as sinonasal carcinosarcomas. The proliferative potential of both epithelium and mesenchyme is evident due to the high pattern of Ki-67 labelling. Thus, it is possible to characterise a specimen as sinonasal carcinosarcoma and differentiate it from other malignancies, such as oesophageal carcinoma and teratosarcoma [[13], [14], [15],^20^,^21^].

Cases treated with surgery and adjuvant radiotherapy are reported with better prognoses. Radiation is conducted after surgery to prevent local recurrence; however, it is not possible to accurately estimate the survival of patients with carcinosarcoma, especially in the maxillary sinuses. Chemotherapy is suggested as a possible treatment, albeit its use is demographically variable [1,4]. In the case presented, probably due to the invasiveness of the case, the surgical treatment regime combined with radiotherapy and chemotherapy did not prevent the patient from coming to death. Which again highlights the importance of an early diagnosis.

In a case reported in the literature, the patient was initially treated with radiotherapy and chemotherapy due to the size of the lesion and then submitted to surgical treatment [13]. In the SEER database survey, surgery associated with radiotherapy was the most frequent [2] and considered more effective treatment by Jeong-Ki Moon [1], while surgery followed by chemotherapy was assumed to provide a better prognosis by Hisham B. Alem.

## 4 Conclusion

Besides being rare and extremely aggressive, sinonasal carcinosarcomas present worse patient survival when compared to other carcinosarcomas in the head and neck region. Thus, this report may contribute to a better understanding of this tumour behaviour.

## Declaration of Competing Interest

All authors declare no conflict of interest related to this manuscript.

## Funding

This manuscript received no specific grant from funding agencies in the public, commercial, or not-for-profit sectors.

## Ethical approval

We declare that our institution does not require ethical approval of clinical case reports.

## Consent

The patient's consent form was obtained.

## Authors’ contributions

JJVP and JTCJ contributed in conceptualization. ATLM, BTCS contributed in study concept and design. ATLM, ELSC contributed in writing the paper. SMAJ contributed in diagnosis histopathological. FSNF, JTCJ and BTCS contributed in surgical management and case record. VAMM performed and interpreted the immunohistochemistry method, which is essential for the diagnosis of the case presented.

## Registration of research studies

None.

## Guarantor

The guarantor of this work, Joao de Jesus Viana Pinheiro, accept full responsibility for the study and the conduct of the study, had access to the data, and controlled the decision to publish.

## Provenance and peer review

Not commissioned, externally peer-reviewed

## Figures statement

We declare that the images in this paper do not reveal information about the patient or the authors.
